# The comparison between foam rolling either combined with static or dynamic stretching on knee extensors’ function and structure

**DOI:** 10.5114/biolsport.2023.119987

**Published:** 2022-11-16

**Authors:** Kazuki Kasahara, Andreas Konrad, Riku Yoshida, Yuta Murakami, Shigeru Sato, Ryoma Koizumi, David G Behm, Masatoshi Nakamura

**Affiliations:** 1 Institute for Human Movement and Medical Sciences, Niigata University of Health and Welfare, Niigata, Japan; 2 Institute of Human Movement Science, Sport and Health, University of Graz, Graz, Austria; 3 Department of Physical Therapy, Niigata University of Health and Welfare, Niigata, Japan; 4 School of Human Kinetics and Recreation, Memorial University of Newfoundland, St. John’s, Newfoundland and Labrador, Canada; 5 Faculty of Rehabilitation Sciences, Nishi Kyushu University, 4490–9 Ozaki, Kanzaki, Saga, 842–8585, Japan

**Keywords:** Isometric contraction, Concentric contraction, Warm-up routine, Range of motion

## Abstract

Static stretching (SS) and dynamic stretching (DS) in combination with foam rolling (FR) have been attracting attention as warm-up routines in sports. However, the combined and intervention order effects of SS or DS and FR on flexibility, muscle strength, and jump performance are still unclear. Therefore, this study aimed to compare the combined effects of FR and SS or DS with the various intervention orders (i.e., SS + FR, DS + FR, FR + SS, DS + FR) on the function and properties of the knee extensors. Using a crossover, random allocation design, 17 male university students (21.0 ± 1.1 y) performed four conditions combining FR and SS or DS. The measurement included knee flexion range of motion (ROM), pain pressure threshold (PPT), tissue hardness, maximum voluntary isometric contraction (MVC-ISO), maximum voluntary concentric contraction (MVC-CON) torque, and single-leg countermovement jump (CMJ) height of the knee extensors. All interventions significantly (p < 0.01) increased knee flexion ROM (SS + FR: d = 1.29, DS + FR: d = 0.45, FR + SS: d = 0.95, FR + DS: d = 0.49), and significantly (p < 0.01) decreased tissue hardness (SS + FR: d = -1.11, DS + FR: d = -0.86, FR + SS: d = -1.29, DS + FR: d = -0.65). There were no significant changes in MVC-ISO, MVC-CON, and CMJ height in all conditions, but a near significant, small magnitude (p = 0.056, d = -0.31) decrease of MVC-ISO was observed in the FR + SS condition. Our results showed that all the combinations of SS or DS and FR effectively decreased tissue hardness and increased ROM without decreasing muscle strength. Also, effect sizes indicated the largest increase in ROM and decrease in tissue stiffness after SS + FR without decreasing muscle strength and jump performance.

## INTRODUCTION

The warm-up before exercise typically consists of aerobic exercise and stretching, i.e., static stretching (SS) and dynamic stretching (DS) [1]. Concerning SS, previous studies showed that a single bout of SS can increase the joint range of motion (ROM) [1, 2], corresponding with a decrease in muscle stiffness [3, 4]. However, further evidence showed that more than 60 seconds of SS per muscle group in isolation (no additional warm-up activities) could decrease muscle strength and performance [1, 5, 6]. On the other hand, previous studies on DS reported no adverse effect on muscle strength or sprinting ability [1, 7] but a similar increase in flexibility compared to SS [8, 9], although no consensus has been reached [10–12]. According to these findings, it can be suggested to use DS rather than SS as a warm-up if the goal is to increase flexibility and sustain performance levels.

In addition, recent studies showed that foam rolling (FR) has been attracting attention as a new warm-up tool due to the increase in ROM, similar to SS and DS [13]. Moreover, two meta-analyses showed that the increase in ROM after FR is similar to stretching [13, 14]. In addition, a further meta-analysis showed a favorable effect on muscle performance after FR compared to SS but no advantage over DS (12). Based on these findings, the authors recommend either DS or FR but not SS as a warm-up tool when the goal is to optimize performance [15].

A meta-analysis comparing the acute effects of some stretching interventions combined with FR on ROM and physical performance showed significantly increased ROM in the combined stretching and FR intervention compared to the control condition [16]. Interestingly, the magnitude of change in performance was similar when FR was performed after stretching compared to stretching alone. On the other hand, when FR was performed before stretching, the effect size of this observation was trivial (ES = 0.17), but the combined application slightly improved performance over stretching alone (P = 0.04). In a report examining the combined and order effects of SS and FR on knee extensors, the combined application of SS and FR increased ROM, while SS followed by FR decreased muscle strength [17].

Furthermore, a recent review comparing the combined effects of DS and FR with those of DS alone concluded that in agility and performance, the combination of DS and FR significantly increased over DS alone [18]. This is consistent with the report [19], which concluded that the combination of DS and FR might significantly increase flexibility, power, and agility. However, few reports have examined the combined effects of DS and FR, and the effects of the intervention order of FR and DS on ROM and performance are unknown.

Based on the previous studies [16, 18, 19], the combined effect of stretching and FR could be greater than SS, DS, and FR intervention alone. These studies suggest that combined stretching and FR could be effective as a pre-exercise warm-up. Still, there might be differences in the effects obtained depending on the intervention order in the combination of stretching and FR, such as the decrease in muscle strength after FR intervention followed by SS intervention [17]. Since the warm-up before exercise is intended to optimize athletic performance [7], it is essential information for athletes and coaches to establish the optimal warm-up method. However, to the best of the authors’ knowledge, no study has compared the effect of the combination and intervention order effect of SS and FR or DS and FR on the same participants in random order in a crossover trial. Therefore, this study aimed to investigate the combined and intervention order effects of SS or DS and FR on knee flexion ROM, muscle strength, tissue hardness, pain pressure threshold (PPT), and single-leg countermovement jump (CMJ) height of the knee extensors. In this study, since the effects of SS and DS to increase ROM are comparable [8], it was expected that the effects of SS + FR and DS + FR to increase ROM would be comparable regardless of the intervention order. Concerning performance, the SS + FR and FR + SS suggest a difference in effect depending on the intervention order [17]. Therefore, we expected that the combination of DS + FR would be optimal as a warm-up, as it is expected to have a beneficial effect regardless of the intervention order.

## MATERIALS AND METHODS

### Experimental set-up

**FIG. 1:**
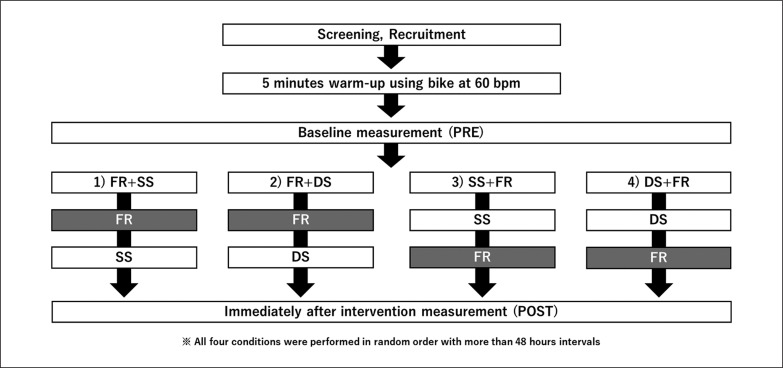
The experimental set-up SS: static stretching, FR: foam rolling, DS: dynamic stretching.

A randomized repeated-measures experimental design was used to compare the order effects of combined anterior thigh SS or DS with FR. The participants were instructed to visit the laboratory four times with a ≥ 48 h break. They were exposed to the following four conditions (Figure 1): FR + SS, FR + DS, SS + FR, and DS + FR. For each SS, DS, and FR, three 60 s bouts were performed on the dominant leg. Outcomes were measured before (PRE) and immediately after the intervention (POST) in each condition. We assessed knee flexion ROM, tissue hardness, PPT, MVC-ISO, maximal concentric (MVC-CON) torque of knee extensors, and unilateral CMJ height in this order, at both PRE and POST.

### Participants

Seventeen healthy, recreationally active males were enrolled (mean ± SD: age, 21.6 ± 1.1 years; height, 170.5 ± 5.8 cm; weight, 66.0 ± 8.6 kg). The participants completed the four conditions described above in random order. Individuals with a history of neuromuscular disease and musculoskeletal injury involving the lower extremities were excluded. The required sample size for a repeatedmeasures two-way analysis of variance (ANOVA) (effect size = 0.40 [large when considering interaction effects for 2-way ANOVAs], α error = 0.05, and power = 0.80) based on our previous study’s ROM results using G* power 3.1 software (Heinrich Heine University, Düsseldorf, Germany) was 14 participants.

For the study, participants were fully informed about the procedures and aims, after which they provided written informed consent. The study complied with the requirements of the Declaration of Helsinki and was approved by the Ethics Committee of the Niigata University of Health and Welfare, Niigata, Japan (Procedure #18615).

### Knee flexion ROM

**FIG. 2:**
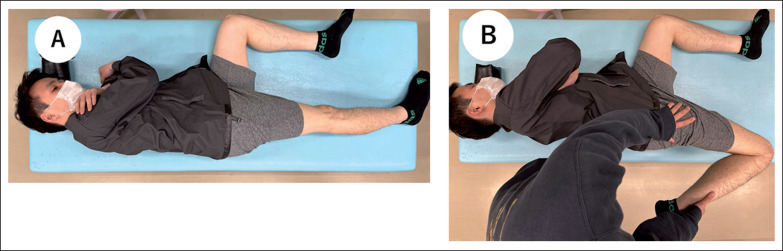
The set-up for knee flexion range of motion (ROM) measurement. The participants lie in a side-lying position with the contralateral hip and knee joints flexed 90° (A). The investigator brought the dominant leg to full knee flexion with the hip joint in a neutral position (B).

Each participant was placed in a side-lying position on a massage bed with the hips as well as the knee of the non-dominant leg flexed at 90° to prevent pelvic movement (Figure 2-A) [20]. A licensed physical therapist, the investigator, brought the dominant leg to full knee flexion with the hip joint in a neutral position (Figure 2-B). A goniometer (MMI universal goniometer Todai 300 mm, Muranaka Medical Instruments, Co., Ltd., Osaka, Japan) was used to measure the knee flexion ROM three times at both PRE and POST in each condition, and the average value was used for further analysis [21].

### Pain pressure threshold (PPT)

PPT measurements were conducted in the supine position using an algometer (NEUTONE TAM-22 (BT10); TRY-ALL, Chiba, Japan). The measurement location was set at the midway of the distance between the anterior superior iliac spine and the dominant side’s superior border of the patella for the rectus femoris muscle. With continuously increasing pressure, the soft tissue in the measurement area was compressed with the metal rod of the algometer. The participants were instructed to immediately press a trigger when pain, rather than just pressure, was experienced. The value read from the device at this time point (kilograms per square centimeter) corresponded to the PPT. Based on previous studies [22, 23], the mean value (kilograms per square centimeter) of three repeated measurements with a 30-s interval was taken for data analysis at both PRE and POST in each condition.

### Tissue hardness

Tissue hardness was measured using a portable tissue hardness meter (NEUTONE TDM-N1; TRY-ALL Corp., Chiba, Japan). The participant’s measurement position and posture were similar to PPT measurements. This tissue hardness meter measured the penetration distance until a 14.71 N (1.5 kgf) pressure was reached [24]. The participants were instructed to relax while tissue hardness measurements were assessed three times at PRE and POST in each condition. The average value was used for further analysis.

### Maximal voluntary isometric (MVC-ISO) and concentric contractions (MVC-CON)

MVC-ISO of the dominant leg’s knee extensors was measured at 70° knee flexion using an isokinetic dynamometer (Biodex System 3.0, Biodex Medical Systems Inc., Shirley, NY, USA). The participants sat on the dynamometer chair adopting an 80° hip flexion angle, with adjusted Velcro straps fixed over the exercised limb’s trunk, pelvis, and thigh. The participants were instructed to contract the knee extensors for three seconds maximally. Two repetitions with a 60-s rest between trials were performed at both PRE and POST [20]. The mean of both repetitions was used for further analysis. MVC-CON was measured at an angular velocity of 60° between 20° and 110° knee flexion. From the three trials performed at both PRE and POST in each condition, the highest value was analyzed. During all tests, strong verbal encouragement was provided to elicit maximal effort.

### Unilateral Countermovement jump (CMJ) height

Unilateral CMJ height was calculated from flight time using a contact mat (Jump mat system; 4Assist, Tokyo, Japan). The participants started with the foot of the dominant leg on the mat with their arms crossed in front of their chest. The participants were instructed to dip quickly (eccentric phase) from this position, reaching a self-selected depth to jump as high as possible in the next concentric phase. Landings were performed on both feet. The knee of the non-involved leg was held at approximately 90° flexion. After three familiarization trials, three maximal unilateral CMJ were conducted at both PRE and POST in each condition, and the greatest vertical jump height was utilized for further analysis [25].

### Test-retest reliability of measurements

The test-retest reliability of knee ROM, PPT, tissue hardness, MVC-ISO, MVC-CON, and CMJ height for 12 healthy men (age, 21.8 ± 1.3 years; height, 169.9 ± 5.9 cm; weight, 67.0 ± 9.6 kg) was determined using the coefficient variation (CV) and intraclass correlation coefficient (ICC), with 10 minutes rest interval between two measures in the similar protocol in this study.

### Foam rolling (FR)

The participants were instructed on how to use the foam roller (Stretch Roll SR-002, Dream Factory, Umeda, Japan) by a physical therapist. For familiarization, they were allowed to practice using the foam roller three to five times on the non-dominant leg (non-intervention leg) immediately before the FR intervention in order to verify that the participants were able to perform the FR intervention at the specified velocity and location. The participants performed three 60-s bouts of FR with a 30-s rest between sets. The participants were instructed to be in the plank position with the foam roller at the most proximal portion of the quadriceps of the dominant leg only. We defined one cycle of FR as one distal rolling plus one subsequent proximal rolling movement. FR velocity was set at 30 cycles per 60-s (90 cycles in three sets) and controlled using a metronome (Smart Metronome; Tomohiro Ihara, Japan). This procedure followed the recommendations of Behm et al. [26] to maximize the increase in ROM. The participants were asked to place as much body mass on the roller as tolerable.

### Static stretching (SS)

SS was conducted similarly to the knee flexion ROM assessment (side-lying position). A well-trained investigator conducted three 60-s bouts with a 30-s rest interval. The participants were instructed to be relaxed and keep their torso upright during stretching.

### Dynamic stretching (DS)

**FIG. 3:**
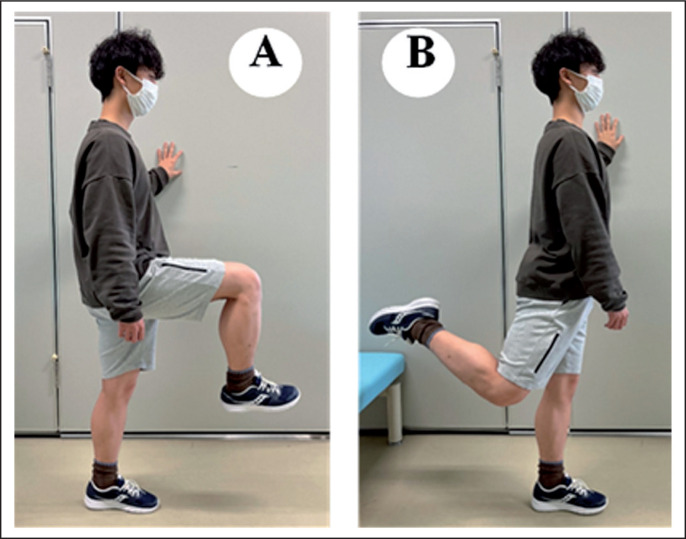
The dynamic stretching (DS) intervention method. The participants performed the DS in the standing position. The participant’s knee and hip joints were initially positioned at 90 degrees flexion (A) and then moved dynamically to a hip extension movement over a 1 second period (B), and then returned to the initial position over another 1-second duration (A).

The participants performed the DS in the standing position. The participant’s knee and hip joints were initially positioned at 90 degrees flexion (Figure 3-A) and then moved dynamically to a hip extension movement over a 1-s period (Figure 3-B), and then returned to the initial position over another 1-s duration (Figure 3-A). As with FR, the metronome was used to perform three 60-s exercises with 30-s rest intervals. The participants were instructed to perform the exercise through a full range of motion under control without using recoil or bouncing to distinguish this from ballistic stretching.

### Statistical analysis

SPSS (version 25.0; IBM Corp., Armonk, NY, USA) was used for statistical analyses. To verify the consistency of PRE values, PRE values were tested among all conditions using a one-way ANOVA. A repeated-measures two-way ANOVA (time [PRE vs. POST] × conditions [FR + SS vs. FR + DS vs. SS + FR vs. DS + FR]) was used to identify interactions and main effects. If the interaction effect was significant, a post-hoc analysis was conducted using paired t-tests with Bonferroni correction on each condition to determine the difference between PRE and POST values. Also, POST values were tested among all conditions using paired t-tests with Bonferroni correction. Effect sizes (ES) were calculated as the mean difference between PRE and POST divided by the pooled PRE and POST standard deviation (SD). An ES of 0.00–0.19 was considered trivial, 0.20–0.49 was small, 0.50–0.79 was moderate, and ≥ 0.80 was large [27]. The significance level was set at 5%. All results are shown as mean ± SD.

## RESULTS

### Test-retest reliability of measurements

The CVs of measurements for knee ROM, PPT, tissue hardness, MVC-ISO, MVC-CON, and CMJ height were 0.2 ± 0.1%, 6.6 ± 3.9%, 1.7 ± 1.3%, 1.5 ± 1.4%, 2.2 ± 2.1%, 1.8 ± 1.9%, respectively, and the ICC (1, 1) for measurements were 0.993, 0.993, 0.970, 0.966, 0.939, and 0.963, respectively.

### Comparison between PRE values among the four conditions

There were no significant differences in all PRE variables among the four conditions.

### Changes in knee flexion ROM, PPT, tissue hardness MVC-ISO, MVC-CON, and CMJ height

Table 1 shows the changes in knee flexion ROM, PPT, tissue hardness, MVC-ISO, MVC-CON, and CMJ before and after the intervention. Significant interaction effects in knee flexion ROM (F = 13.2, p < 0.01, η_p_^2^ = 0.38), MVC-ISO (F = 2.9, p = 0.042, η_p_^2^ = 0.12) and PPT (F = 3.2, p = 0.03, η_p_^2^ = 0.13) were revealed. Post-hoc test results showed that knee flexion ROM significantly increased in all conditions. MVC-ISO experienced a near significant decrease (FR + SS: p = 0.056, d = -0.31), compared to non-significant changes for FR + DS: (p = 0.46, d = 0.17), SS + FR (p = 1.00, d = 0.01), and DS + FR (p = 1.00, d = 0.07). However, there were no significant differences in POST values in MVC-ISO among the four conditions. PPT significantly increased (p < 0.01) in FR + SS (d = 0.33), FR + DS (d = 0.61), and DS + FR (d = 0.82) conditions, but SS + FR condition showed no significant change (p = 0.24, d = 0.31).

**TABLE 1:** The changes (mean ± SD) in knee flexion range of motion (ROM), pain pressure threshold (PPT), tissue hardness, maximal voluntary isometric contraction (MVC-ISO), maximal voluntary concentric contraction (MVC-CON) torques, and counter-movements jump (CMJ) height before and after the intervention. The two-way repeated ANOVA results (T: time effects, C × T: condition × time interaction effects; P- and F- value) and partial η^2^ (η_p_^2^) are shown in the right column.

	FR + SS		FR + DS		SS + FR		DS + FR		ANOVA results
	PRE	POST	PRE	POST	PRE	POST	PRE	POST	P value, F value, η_p_^2^
Knee flexion ROM (degrees)	132.7 ± 6.9	138.9 ± 6.1*	133.2 ± 5.8	136.2 ± 6.5*	132.8 ± 5.0	138.6 ± 3.9*	132.1 ± 5.0	134.4 ± 5.1*^†‡^	T: F = 256.0, p < 0.01, η_p_^2^ = 0.80
	d =	0.95	d =	0.49	d =	1.29	d =	0.45	C × T: F = 13.2, p < 0.01, η_p_^2^ = 0.38

PPT (kg)	2.8 ± 1.3	3.2 ± 1.2*	2.5 ± 1.1	3.1 ± 1.0*	2.9 ± 1.4	3.3 ± 1.8	2.5 ± 1.0	3.5 ± 1.5*	T: F = 49.9, p < 0.01, η_p_^2^ = 0.44
	d =	0.33	d =	0.61	d =	0.23	d =	0.82	C × T: F = 3.1, p = 0.03, η_p_^2^ = 0.13

Tissue hardness (N)	19.0 ± 3.0	15.6 ± 2.3*	18.9 ± 3.1	16.8 ± 3.4*	18.4 ± 2.3	15.0 ± 3.9*	17.9 ± 2.5	15.9 ± 2.2*	T: F = 124.2, p < 0.01, η_p_^2^ = 0.66
	d =	-1.29	d =	-0.65	d =	-1.11	d =	-0.86	C × T: F = 2.6, p = 0.06, η_p_^2^ = 0.11

MVC-ISO (Nm)	216.3 ± 42.4	203.8 ± 37.9	210.9 ± 34.5	216.7 ± 34.0	216.4 ± 42.7	216.8 ± 48.3	216.5 ± 36.7	219.0 ± 39.4	T: F = 0.2, p = 0.687, η_p_^2^ = 0.003
	d =	-0.31	d =	0.17	d =	0.01	d =	0.07	C × T: F = 2.9, p = 0.042, η_p_^2^ = 0.12

MVC-CON (Nm)	172.7 ± 25.0	165.5 ± 25.6	173.2 ± 23.5	177.3 ± 26.1	174.1 ± 31.1	173.3 ± 35.6	175.6 ± 24.8	177.3 ± 25.4	T: F = 0.1, p = 0.74, η_p_^2^ = 0.002
	d =	-0.29	d =	0.17	d =	-0.02	d =	0.07	C × T: F = 2.1, p = 0.12, η_p_^2^ = 0.09

CMJ height (cm)	21.1 ± 3.8	20.7 ± 3.7	23.2 ± 6.4	22.1 ± 3.6	21.6 ± 3.7	21.1 ± 4.0	21.6 ± 3.2	22.2 ± 3.8	T: F = 1.2, p = 0.27, η_p_^2^ = 0.019
	d =	-0.11	d =	-0.23	d =	-0.13	d =	0.17	C × T: F = 1.1, p = 0.34, η_p_^2^ = 0.05

* : Significantly (P < 0.05) different from the PRE-value.

† : Significantly different compared to POST values for FR + SS conditions.

‡ : Significantly different compared to POST values for SS + FR conditions. SS: static stretching, DS: dynamic stretching, FR: foam rolling

Additionally, there were no significant interaction effects for MVC-CON, CMJ, or tissue hardness. However, there was a main effect of time on tissue hardness (F = 124.2, p < 0.01, η_p_^2^ = 0.66), which demonstrated that tissue hardness decreased after the intervention compared to PRE values in all conditions.

## DISCUSSION

Previous studies have examined the effects of SS and FR and DS and FR in combination [17–19]. However, to the best of our knowledge, this was the first study to compare the combined effects of SS or DS and FR with a crossover design. The results showed a significant ROM increase and tissue hardness decrease in all conditions. Interestingly, the effect size of knee flexion ROM changes was greater for both FR + SS and SS + FR than for FR + DS or DS + FR conditions. Although there was no significant change in muscle strength in all interventions, there was a near significant decrease in muscle strength in the SS + FR condition (p = 0.056, d = -0.31).

As shown in Table 1, all interventions in this study increased knee flexion ROM significantly. This result supports the results of Nakamura et al. [17], who investigated the combined effect of SS and FR, and Hsu et al. [19], who investigated the combined effect of DS and FR. Previous studies have suggested that changes in stretch tolerance and passive stiffness are involved in the increase in ROM after stretching interventions [28]. Indeed, a previous study showed that increased ROM with stretching could be associated with a change in stretch tolerance [29]. Similarly, stretch tolerance could contribute to change in ROM after a single FR intervention [30–32]. Concerning this study, the detailed mechanism of the increased knee flexion ROM is unknown, but changes in stretch tolerance might be involved in the increase in ROM.

Although not statistically significant, the effect sizes of DS + FR and FR + DS on ROM were small (d = 0.45 and d = 0.49, respectively), while SS + FR and FR + SS showed large effect sizes (d = 1.29, and d = 0.95, respectively). Regarding the difference in the combined effect of SS or DS with FR, several studies indicate greater ROM with SS than DS [33–38]. However, others have concluded that SS and DS have similar [8, 9] or greater [10, 11] acute increases in ROM as static stretching. Therefore, there is no consensus about the increase in ROM after SS or DS, but the results of this study indicated that SS could increase knee flexion ROM to a greater extent than DS when combined with FR. This could be related to the difference in the change in passive stiffness between SS and DS interventions. Mizuno et al. [39] showed that increased ROM after SS could be related to decreased passive stiffness and changes in stretch tolerance. Although SS interventions could decrease passive stiffness [3, 4, 40], a previous study showed that DS interventions did not produce significant changes in passive stiffness [41]. Therefore, the detailed mechanism of the difference in the increase in knee flexion ROM after combining SS or DS with FR is unclear. Still, the combination of SS and FR could have a greater effect on reducing passive stiffness than the combination of DS and FR, resulting in a larger increase in knee flexion ROM.

Interestingly, there was a significant increase in PPT in the FR + SS, FR + DS, and DS + FR conditions but not in the SS + FR condition (p = 0.16, d = 0.31). A previous study showed that mechanical stimulation by FR intervention could reduce pain perception [42]. Thus, 180-s of FR intervention could have increased PPT in combined FR and SS or DS. In addition, tissue hardness was significantly decreased in all conditions. The effect sizes on tissue hardness were greater for the combination of SS and FR (SS + FR: d = -1.11, FR + SS: d = -1.29) than for the combination of DS and FR (DS + FR: d = -0.86, FR + DS: d = -0.65). As mentioned earlier, SS intervention reduced passive stiffness [3, 4, 40], whereas DS could induce no change in passive stiffness [41]. Additionally, it was speculated that FR could induce thixotropic changes in intramuscular hyaluronic acid and alter muscle viscoelasticity [43]. Therefore, the combination of FR and SS could have produced a greater reduction in tissue hardness than the combination of FR and DS.

This study showed that MVC-ISO torque did not change in all conditions, but there was a near-significant, small magnitude decrease in the SS + FR condition (p = 0.056, d = -0.31). Previous studies pointed out that a longer duration than 60 seconds of SS could produce performance impairments [1, 6, 7]. Neurological and morphological factors have been considered as possible mechanisms [5]. Previous studies have reported that stretching may induce changes in persistent inward current (PIC) [5] and muscle spindle sensitivity [44], which could have adverse effects on muscle strength. However, the previous study suggested that FR after SS restores motoneuron excitability, resulting in recovery from a decrease in muscle strength [17]. Therefore, MVC-ISO could not change in the SS + FR condition.

On the other hand, DS is considered to increase muscle strength and/or athletic performance [45]. However, the effects of FR on muscle strength and jumping height have been shown to be negligible [26]. Therefore, we hypothesized that the combination of DS and FR would increase muscle strength and jump performance. Contrary to our expectations, however, there were no significant differences between conditions in MVC-ISO, MVC-CON, and CMJ height in the combined DS and FR conditions. These results were inconsistent with a previous study [19], in which combined DS and FR resulted in a significant increase in broad jump and medicine ball chest throw distance. This difference in results could be due to the time of DS intervention. The duration of the DS intervention in this study was 180 seconds, but eight types of DS were performed for a total of 480 seconds in the previous study [19]. DS has been shown to potentially improve performance with longer stretch times [46, 47]. Therefore, the combination of DS and FR intervention could induce increases in muscle strength and athletic performance when a longer duration of DS intervention time is applied than in the present study. Still, it is also possible that greater muscle strength and performance improvement effects may not occur when used in combination with FR. Also, no significant changes were observed in MVC-CON and CMJ height in all conditions. The previous study has shown that isometric contractions could promote recovery of muscle spindle sensitivity [44]. Since MVC-CON and CMJ were measured after MVC-ISO in this study, MVC-ISO measurement could facilitate the recovery of muscle spindle sensitivity altered by SS, resulting in no changes in MVC-CON and CMJ height in the combination of SS and FR.

There were limitations in this study. There was no control condition in this study. However, we have confirmed that the test-retest reliability of measurements is very high. Therefore, we believe that the results of this study reflect the effects of the interventions. The participants performed three 60-s bouts of FR or stretching intervention with a 30-s rest between sets, but the rest interval might affect the intervention effect. Also, the participants were recreationally active but not athletes. Previous studies investigated the effect of warmup routine using FR and stretching intervention on athletic performance in well-trained tennis players [19, 48]. Still, it is unclear whether the results of this research could be applied to athletes. Thus, further study is needed to investigate the effect of different rest intervals of FR and stretching interventions on ROM and athletic performance in athletes to establish an effective pre-exercise warm-up in a sports setting.

### Practical implications

As a pre-exercise warm-up, a combination of SS and FR is recommended if the goal is only to increase ROM without necessarily improving performance. On the other hand, the combination of DS and FR and FR after SS intervention conditions showed no change in muscle strength and jump performance. Therefore, the SS + FR condition is the best approach when the goal is to increase ROM while maintaining muscle strength and jump performance.

## CONCLUSIONS

This study aimed to compare the combined and order effects of SS or DS and FR to establish the optimal warm-up routine in sports. All interventions showed significant changes in knee flexion ROM, PTT, and tissue hardness. However, the changes in knee flexion ROM and tissue hardness after the combination of SS and FR could be superior to the combination of DS and FR regardless of an intervention order.
